# Did Quinine Kill Them?

**Published:** 1891-04

**Authors:** J. R. G. Howell

**Affiliations:** Dothan, Ala.


### DID QUININE KILL THEM?

J. R. G. HOWELL, M. D., DOTHAN, ALA.

In the February number of the JOURNAL, page 712, I notice an article by E. H. Martin, M. D., of Hillhouse, Miss., commenting on and criticising my nomenclature and treatment of malarial hæmoglobinuria in the December number of the JOURNAL, page 609. His criticism of the term hæmorrhagic malarial fever is well sustained, for the term is an inappropriate one. But as I am not the author of it, I am not responsible for its origin. The criticism of the use of quinine in the treatment of said disease is the part of his article I am going to answer. Dr. M. censures me for not being able to see that quinine is contra-indicated in malarial hæmoglobinuria. I wish to show:

1. That quinine is not the cause of malarial hæmoglobinuria.

2. That quinine was not the cause of the fatal results in cases I., II., III. and IV., reported by Dr. M., as he attempts to show.

3. That calomel was the drug that killed them.

It is a fact that quinine is sometimes, but *not always* given previous to attacks of said disease. Also are calomel, arsenic, salicylic acid and many other remedies. Why? Because the patients are in a malarial district. Now is this any reliable reason for calling the ill that may occur in the future, a case of quinine hæmoglobinuria or calomel hæmoglobinuria, or any other name that would imply that a given drug was the cause of the disease. The patient is in a malarial district, under malarial surroundings, and breathing malaria. This is plain but strange evidence that malaria is the cause.

Dr. M. makes inquiry just far enough to find out that his patient has been taking quinine, and there he ceases his inquiry, satisfied that he had a case of "quinine hæmoglobinuria."

The following table of Feraud will prove very conclusively the second and third propositions of my article. For this table I am indebted to Dr. H. McHatton, of Macon, Ga.:

| | Cases. | Deaths. | Per cent. | |
|---|---|---|---|---|
| a | 71 | 22 | 31 | Quinine in very small doses. Calomel purgative. |
| d | 11 | 4 | 36 | Quinine very small doses. Calomel and other purgatives base of treatment. |
| e | 42 | 13 | 31 | Quinine very small doses. Calomel and other purgatives base of treatment. |
| f | 30 | 9 | 30 | Quinine very small doses. Calomel and other purgatives base of ment. |
| b | 40 | 8 | 20 | Quinine in medium doses. Calomel in small doses. |
| c | 29 | 5 | 17 | |
| g | 27 | 3 | 17 | |
| h | 18 | 2 | 11 | Quinine in large doses. |
| i | 18 | 0 | 0 | |

As before said, this table shows that quinine in large doses is the remedy for the disease in question. It also shows that calomel is the poison. We can see the same truth very prominent in the eleven cases reported by Dr. Martin. We see that in cases I., II., III. and IV., he gave both quinine and calomel. They all died. Now, why does Dr. M. charge all his bad luck up to quinine? Why charge any of it to that grand old remedy? In case I., we see on the 11th, Dr. M. gave calomel in four grain doses, repeated every hour until six doses were given! We also see on the 14th, his patient "flickered out," as he describes it. We also see that patients II., III. and IV. had calomel in alarming quantities (for calomel is always alarming in malarial hæmaturia), and they all died promptly. He acknowledges, by accusing me of the same error, the fact that he gave alternately a poison and an antidote, but alas! his poison, we see, was too powerful.

He censures me for not seeing that I was killing my patient with quinine. I gave quinine without calomel, and that as well as all the other cases coming under my treatment recovered as speedily as heart could wish for.

Dr. M. treated his remaining seven cases without either quinine or calomel, and as might have been expected, he lost some and some *got well.* Now I am perfectly willing for Dr. M. to name the disease "Lysæmia," provided he will notice the use of quinine and the abuse of calomel in malarial hæmoglobinuria.

